# Power, measurement error, and pleiotropy robustness in twin-design extensions to Mendelian Randomization

**DOI:** 10.21203/rs.3.rs-3411642/v1

**Published:** 2023-10-14

**Authors:** Luis FS Castro-de-Araujo, Madhurbain Singh, Yi Zhou, Philip Vinh, Hermine HM Maes, Brad Verhulst, Conor V Dolan, Michael C Neale

**Affiliations:** Virginia Commonwealth University; Virginia Commonwealth University; Virginia Commonwealth University; Virginia Commonwealth University; Virginia Commonwealth University; Texas A&M University; Vrije Universiteit. Amsterdam; Virginia Commonwealth University

**Keywords:** causality, pleiotropy, twin design, Mendelian randomization

## Abstract

Mendelian Randomization (MR) has become an important tool for causal inference in the health sciences. It takes advantage of the random segregation of alleles to control for background confounding factors. In brief, the method works by using genetic variants as instrumental variables, but it depends on the assumption of exclusion restriction, i.e., that the variants affect the outcome exclusively via the exposure variable. Equivalently, the assumption states that there is no horizontal pleiotropy from the variant to the outcome. This assumption is unlikely to hold in nature, so several extensions to MR have been developed to increase its robustness against horizontal pleiotropy, though not eliminating the problem entirely (Sanderson et al. 2022). The Direction of Causation (DoC) model, which affords information from the cross-twin cross-trait correlations to estimate causal paths, was extended with polygenic scores to explicitly model horizontal pleiotropy and a causal path (MR-DoC, Minică et al 2018). MR-DoC was further extended to accommodate bidirectional causation (MR-DoC2 ; Castro-de-Araujo et al. 2023). In the present paper, we compared the power of the DoC model, MR-DoC, and MR-DoC2. We investigated the effect of phenotypic measurement error and the effect of misspecification of unshared (individual-specific) environmental factors on the parameter estimates.

## Introduction

A long-standing challenge in epidemiology has been to infer causality from correlational data in observational studies. For many scientists, correlational studies are starting points for exploring the causal associations between variables. However, by themselves, correlations are insufficient to identify causality, due to the potential existence of background confounding and ambiguous direction of causality.

The primary alternative to observational studies is the randomised controlled trial (RCT), in which study participants are randomly allocated to treatment/experimental and control groups. This approach averages the effects of any confounders equally among the groups, so that any difference in the outcome can be attributed to the intervention. However, RCTs are not always feasible. Difficulties may arise due to ethical considerations, e.g., when the aim is to estimate the causal effects of putative risk factors on disease outcomes, as it is fundamental to not harm participants. This is particularly problematic in psychiatry, for example, when working with samples of children, which hinders intervention evaluation.

Mendelian randomization (MR) can be used to investigate causality in cases where RCTs are not feasible or ethical. It is based on Mendel’s laws of inheritance of segregation and independent assortment. Specifically, it uses the randomization that happens in meiosis, when genetic information is shuffled between chromosomes (crossing-over) and these chromosomes then form the gametes (Madole and Harden 2022). Genetic variants associated with phenotypic exposures are identified in large scale genome-wide association studies (GWAS) and meta-analyses. These genetic variants, or weighted combinations thereof, are potentially useful instrumental variables. Instrumental variables are variables correlated with the predictor, but only indirectly affect the outcome, and are commonly used in econometrics (Evans and Davey Smith 2015; Sanderson et al. 2022).

Several key assumptions are involved in causal inference based on MR (Sanderson et al. 2022). First, the strength of the association between the genetic variant(s) and the exposure must be sufficient, which is known as the *relevance assumption*. Second, MR is based on the assumption that the instrument’s effect on the outcome is completely mediated by the exposure (known as the *exclusion restriction* assumption).. In genetic studies this assumption is known as no *horizontal pleiotropy*. This assumption may not hold, given that GWAS have shown that the same variant may influence multiple traits, sometimes affecting both exposure and outcome, i.e., if the exposure acts as a mediator between the variant and the outcome then it will *not* be a case of horizontal pleiotropy (Verbanck et al. 2018; Jordan et al. 2019). Horizontal pleiotropy, the **direct effect** of the instrument on both the exposure and the outcome, violates the assumption of exclusion restriction in MR.

**Figure 1:**
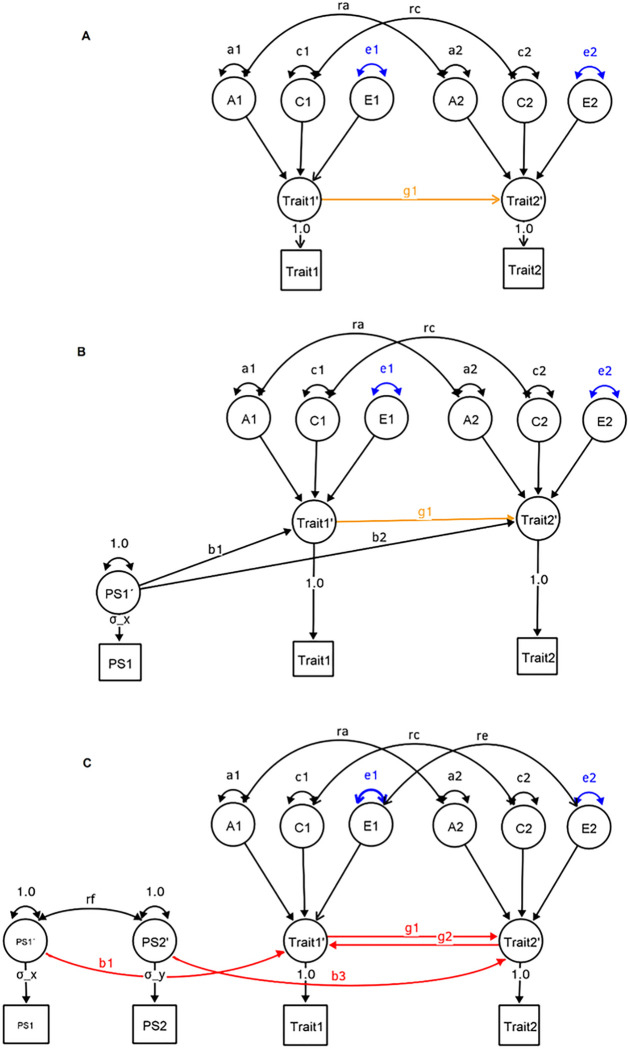
DoC (A), MR-DoC (B), and MR-DoC2 (C) model specifications for a single member of a twin pair. The genetic cross-twin correlations are 1 for MZs and 0.5 for DZs and the shared environmental variance cross-twin correlations are 1 MZs and DZs (not shown). They include the effects of additive genetic (A), common environment (C) and specific environment (E) factors for both Trait 1 and Trait 2, and their effects may correlate (parameters *ra, rc*, and *re*). Path labels in red are important to the model’s power, those susceptible to measurement error in blue, and in orange are those that are both susceptible to measurement error and are important to the model’s power. The latent variables Trait1’ and Trait2’ are not required for identification, but are kept to be coherent to Castro-de-Araujo et. al. (2023) paper, to emphasize the scaling solution of PS1’ and PS2’, and to point that one possible extension of these models is the use of multiple indicators.

Several solutions have been proposed to detect and/or accommodate horizontal pleiotropy in causal inference based on MR (Sanderson et al. 2022). One can use methods that relax this assumption, and only require the instrument strength to be independent of the direct effect of the exposure on the outcome (Bowden et al. 2015), or one can triangulate results from different MR methods to confirm that the strength and direction of the causal signal are consistent over tests (Burgess et al. 2020). Alternative methods that integrate MR in the twin-design were proposed to address the restriction assumption (Hwang et al. 2021), notably MR-DoC (Minica et al. 2018), which combines MR with the Direction of Causation (DoC) twin design (Heath et al. 1993; Duffy and Martin 1994). The MR-DoC model includes a (horizontal) pleiotropic path, accommodating a direct relationship between instrumental variable and the outcome, and thus allowing for a test of directional horizontal pleiotropy (path b2, Fig. 1B). MR-DoC was extended to accommodate bidirectional causation (Castro-de-Araujo et al. 2023; MR-DoC2, Fig. 1) by adding an additional polygenic score that acts as an instrumental variable for the outcome, thus allowing for estimation of the potential effects of reverse causation (paths b1 and b3, Fig. 1C). Henceforth we refer to the Minica et al. (2018) method as MR-DoC, and the Castro-de-Araujo et al. (2023) method as MR-DoC2.

The DoC model uses cross-twin cross-trait correlations to extract information on possible causal paths between two phenotypes. However, it is known to have the following limitations. First, differences in reliability of the variables in the model may bias causal inference estimates (Heath et al. 1993; Gillespie et al. 2003). Specifically, the more reliable variable is more likely to be identified as the cause of the less reliable variable (Heath et al. 1993; Duffy and Martin 1994). Castro-de-Araujo et al. (2023) reported that this is not an issue in MR-DoC2, but it is unknown whether this is an issue in MR-DoC. Second, both the DoC and MR-DOC models require the assumption that the unshared environmental correlation, the parameter re in Fig. 1, is zero in order to estimate the causal path between the exposure and the outcome. This constraint implies the assumption that unshared environmental influences are not a source of confounding. Violation of this assumption biases the causal estimates in DoC models (Rasmussen et al. 2019), but it is not a problem for MR-DoC2, which explicitly models this type of confounding. For MR-DoC it is unknown if *re ≠ 0* introduces biases to the estimates of interest, in particular the causal path (g1), or other paths (Fig. 1, A).

The statistical power of MR-DoC and MR-DoC2 models has been explored in Minica et al. (2018) and in Castro-de-Araujo et al. (2023), respectively. However, a comparison of the power profiles of the three models (i.e., DoC, MR-Doc, and MR-Doc2) is lacking. While all three models focus on causal inference, the models differ with respect to their assumptions. First, the DoC model can accommodate both unidirectional and bidirectional causation, provided that some of the parameters are fixed. That is, in addition to the two causal paths, only one of three possible sources (A, C, E) of confounding can be accommodated. This implies that of the three correlations, ra, rc, and re, two have to be constrained (to zero). In general, a bivariate ACE model is identified with any three of the five possible paths coefficients (parameters ra, rc, re, g1 and g2; Fig. 1), which connect the two phenotypes, freely estimated (Maes et al. 2021). Second, the MR-DoC model is usually specified with unidirectional causation (as bidirectional causation requires further constraints to the background ACE confounding), and assumes no unshared environmental confounding (*re* = 0, Fig. 1). Third, MR-DoC2 is bidirectional and assumes no direct horizontal pleiotropy (b2 path in MR-DoC is fixed to zero in MR-DoC2). However, MR-DoC2 does accommodate two sources of indirect horizontal pleiotropy (rf*b1 and rf*b3 in Fig. 1). Of note, this type of pleiotropy is effectively equivalent to direct horizontal pleiotropy.

In this paper, we compared the power profiles within and between the three models. We estimated the effect of phenotypic measurement error, and the effect of environmental confounding in DoC and MR-DoC (re ≠ 0). We finally identify situations in which each model performs optimally in terms of power. The outline of this paper is as follows. First, the model specifications will be presented; Second, the simulation designs will be presented; Third, bias due to measurement error will be tested by introducing unreliable phenotypes; Fourth, results from simulations where exclusive environmental confounding (re, in Fig. 1) is fixed to zero, when in fact it is present in the data generation process will be presented. Finally, the models’ power profiles will be reported.

## Methods

### Model specification

Specifications of the three models were reported in the original papers: DoC (Heath et al. 1993; Neale & Cardon 1992), MR-DoC (Minică et al. 2018), and MR-DoC2 (Castro-de-Araujo et al. 2023). However, for the present work we will report on results using the variance component style of the three models (Verhulst et al., 2019), whereas in previous papers the model was specified in path coefficients (Minică et al. 2018; Castro-de-Araujo et al. 2023). The code for each model is publicly available as a function in the umx R package (Bates et al. 2019). The choice for the conversion to variance component style was based on the improved Type I error rates in the test of variance components (Verhulst et al. 2019). This approach is also faster to run, which helps in automation in larger analyses, and is consistent with a recent paper published by our group (Maes et al. 2022).

### Simulation designs

We conducted simulation studies to compare DoC, MR-DoC and MR-DoC2, and to investigate known limitations of the direction of causation (DoC) and MR-DoC twin models. We addressed three issues: (A) the effect of unmodeled phenotypic measurement error on parameter estimates; (B) the effect of misspecification of the models with respect to the parameter re (*incorrectly constrained re = 0*); and (C) power, by assessing the contributions of the individual parameters to the non-centrality parameter in tests of null-hypotheses in each model.

We used exact data simulation to generate raw data given a population covariance matrix. Using this method, we ensure that the observed covariance matrix equals the population covariance matrix exactly. This equality means that when fitting the true model (given that it is identified) the parameter estimates match the population values exactly. Consequently, a hypothesis test based on the likelihood ratio (e.g., fixing a parameter to zero), will produce a non-centrality parameter, which we can use in power calculations (van der Sluis et al. 2008). The method comprises five steps: (1) Choose values for the parameters in the model of interest. (2) Simulate multivariate normally distributed exact data based on the expected model covariance matrices and (zero) means of the monozygotic (MZ) and dizygotic (DZ) twins. To this end we used the function *mvrnorm()* in the R library MASS. We equated the MZ and DZ sample sizes at 1000 pairs (Venables et al. 2002). (3) Fit the true model using maximum likelihood estimation, thus recovering the true parameter values. (4) Fit a false model by imposing the constraint(s) of interest (fixing non-zero parameter to zero), and refitting the model. (5) Extract the non-centrality parameter (NCP), which equals the likelihood ratio test statistic associated with the constraint of interest, and use it to calculate the power to reject the parameter of interest given the given Type I error rate of .05.

The five simulations performed were based on three factorial designs (Table 1), in which the parameters featured as design factors, and the parameter values as the factor levels. The chosen parameter values for the levels were based on levels used in our previous publication (Castro-de-Araujo et al. 2023), which attempts to model phenotypes with broad type heritability estimates from 0.32–0.5 and strong instruments (b1, and b3) between 0.16–0.22. All simulation designs included multivariate models with A, C, and E variances. The levels of the factors (values of parameters) are equal across designs. The number of parameters in each design depends on the number of parameters in each of the models (DoC, MR-DoC, and MR-DoC2), thus design 1 has fewer parameters (g1, ra, rc, re, a1, c1, e1, a2, c2, and e2) than Design 2, which in turn has fewer parameters (b1, b2, g1, ra, rc, re, a1, c1, e1, a2, c2, and e2) than Design 3 (b1, b3, g1,g2, ra, rc, re,rf, a1, c1, e1, a2, c2, and e2), as each was based on the models.

**Figure 2:**
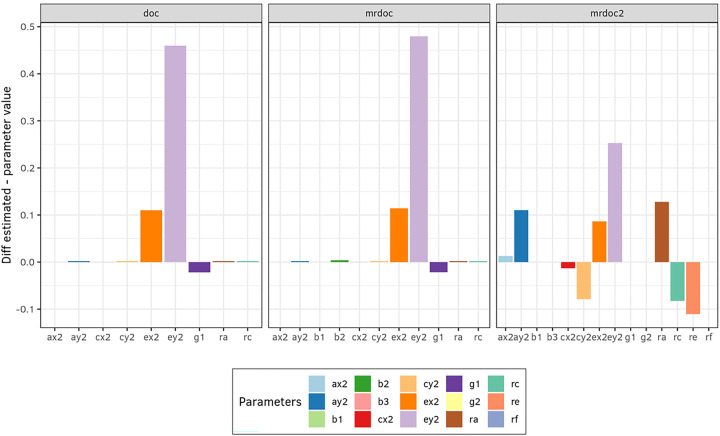
Robustness to measurement error in DoC, MR-DoC, and MR-DoC2. Error in measurement is an important source of bias for the classical DoC model and for MR-DoC. Reliability was set at 90% in the exposure (x) and 70% in the outcome (y) in an exact data simulation (Designs 1, 2, and 3; Table 1). Although the bias is not severe in either case there is underestimation of the causal path from x to y (g1) for DoC and MR-DoC models, which does not happen in the MR-DoC2 model.

We first assessed the effect of unreliability of the phenotypic measurements on the parameter estimated. To do so, we added measurement error to the exposure and to the outcome. The reliabilities of the phenotypes were set to reflect the known shortcoming from the DoC model, where the phenotype is more likely to be identified as the cause of the less reliable phenotype, thus reliability for the exposure was set to .90 and the reliability of the outcome was .70 (Table 1). Bias stemming from the unreliability, was calculated as the mean difference between true parameter values and the parameter estimates averaged across the exact data simulations (Fig. 2).

**Figure 3:**
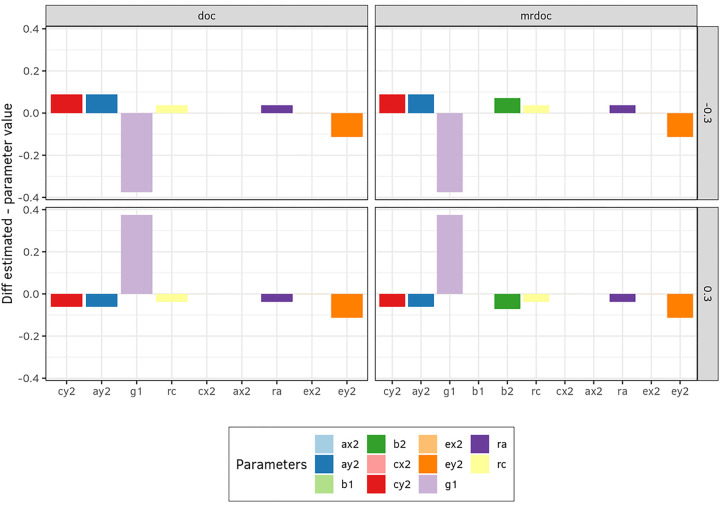
Bias due to *re* misspecification. This was based on designs 1, 2, and 3 (Table 1), and *re* = [+0.3, −0.3] was added to the data generating process and then DoC and MR-DoC models were fitted with *re*= 0 (and not free) . The presence of *re*introduces bias to DoC and MR-DoC notably in *g1*. No bias occurs with MR-DoC2.

In the second simulation, we assessed the effect of unshared environmental confounding on the estimates to evaluate how the unshared environmental correlation (re in Fig. 1) affects the parameter estimates, given that it is fixed to zero to identify both DoC and MR-DoC. In this simulation study, we added the parameter re as a factor, with levels *re=*(0.3 and − 0.3) (Designs 1, 2, and 3), and then fitted models with *re* fixed to equal zero, thus allowing us to address the consequence of violating the assumption *re* = 0, when in truth *re* = 0.3 or re=−0.3. This re level was chosen to be sufficiently large to produce observable bias effects, without being unrealistically large considering that E variance includes measurement error, which does not normally contribute to re. Bias was calculated and plotted in Fig. 3.

### MR-DoC2 as the data generating process

In what follows, we generated data with the exact covariance matrix of the MR-DoC2 model, and we fitted the DoC, MR-DoC, and MR-DoC2 models to the data. The factorial design 3, Table 1, was used. This procedure aimed to evaluate potential biases, given: i) the constraints added to the model (unreliability or re ≠ 0), and ii) the absence of some parameters in DoC and MR-DoC in relation to MR-DoC.

**Figure 4:**
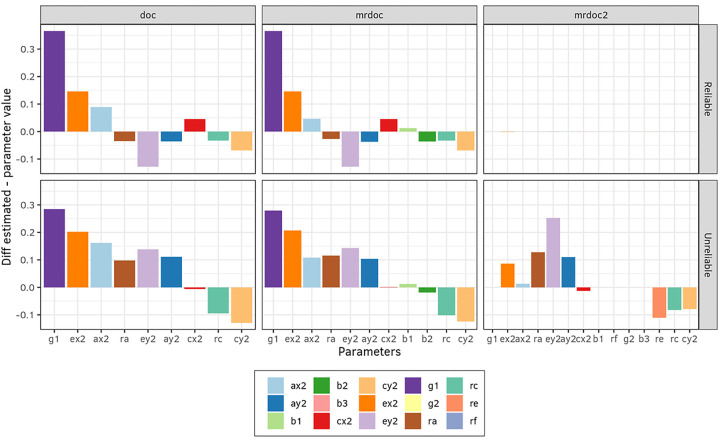
Bias due to unmodeled unreliability. This graph was based on exact data simulation with a reliability of 90% for the exposure and 70% for the outcome, and a panel with the simulation of the reliable phenotype measurement for comparison. MR-DoC2 was used as the data generating process (Design 3, Table 1) and all three models were fitted to the generated data. There are widespread biases in the DoC and in the MR-DoC estimates, with notable overestimation of g1. Contrast this with Figure 2, in which the independent exact data simulation revealed underestimation of g1 in both DoC and MR-DoC.

First, we assessed the consequences of unreliability using this approach, elaborating on the results of the first simulation. Data were generated with the MR-DoC2 model, but the reliability of exposure and outcome were set to 0.90 and 0.70, respectively. DoC, MR-DoC, and MR-DoC2 were then fitted to these data. Whether this resulted in any bias in the parameter estimates of DoC or MR-DoC, was assessed visually in Fig. 4, where differences between parameters and estimates were plotted.

**Figure 5:**
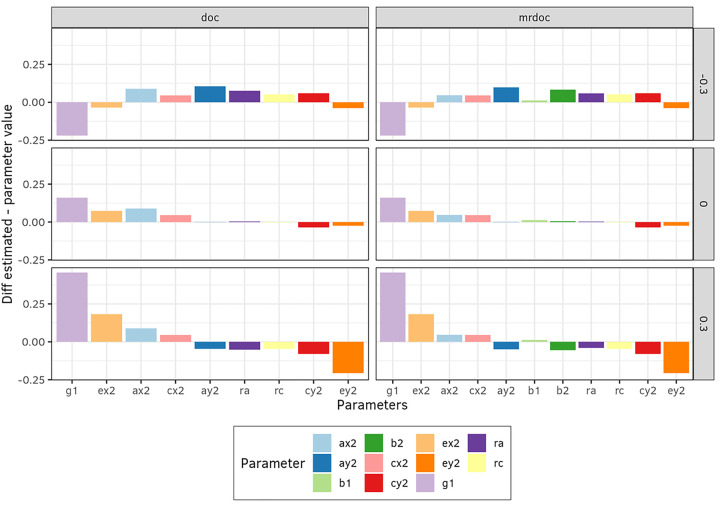
Bias due to the incorrect assumption that *re* = 0. This was based on an exact data simulation, where MR-DoC2 was used as the data generating process (Design 3, Table 1) and each of the three models was then fit to the generated data. In the exact data simulation, an extra factor with two levels [*re* = −0.3, +0.3] was added in the data generating process and then models were fitted with re fixed at zero. The presence of *re* introduces bias to DoC and MR-DoC. No bias occurs with MR-DoC2.

Next, we examined the effect of the violation of the assumption *re* = 0 in DoC and MR-DoC models. An extra factor *re* with two levels *re* = (−0.3 and + 0.3) was added to design 3 (Table 1). Exact data generation was used to simulate data under the MR-DoC2 model, and then all models were fitted to these data. In Fig. 5, bias is plotted for when *re* is either − 0.3 or + 0.3 for all three models. Notice that MR-DoC2 includes *re,* as a freely estimated parameter, whereas it is fixed in DoC and MR-DoC. The difference in covariance when *re* is set to −0.3 in MR-DoC is shown in Table 2 as an example of the magnitude of this bias in the covariance structure.

**Figure 6:**
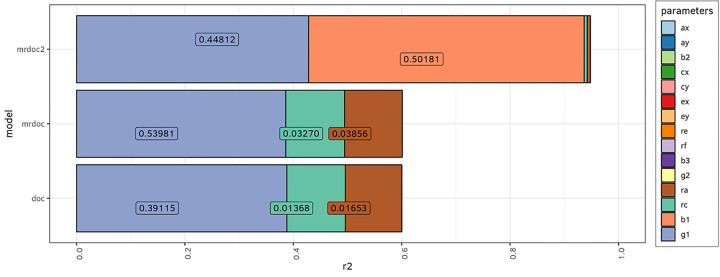
Variation in statistical power (non-centrality parameter; NCP), as a function of model parameter values. Using exact data simulation, the NCP was linearly regressed onto the parameter values to quantify their relative importance.MR-DoC2 was used as the data generating process (Design 3, Table 1) and all three models were then fitted to the generated data. The power test here is for the rejection of the hypothesis that *g1*=0. These are stacked bar plots with the coefficient of each predictor. The total R^2^ for the DoC model was 0.60, 0.60 for MR-DoC, and 0.95 for MR-DoC2. Longer bars mean that *g1* has the largest effect on DoC and MR-DoC power, and that *g1* and *b1* have the largest effects on the MR-DoC2 power to reject g1=0.

The final simulation study aimed at evaluating the contributions of the parameters (*a*_*1*_, *c*_*1*_, *a*_*2*_, *c*_*2*_, *b*_*1*_, *b*_*2*_, *b*_*3*_, *g*_*1*_, *g*_*2*_, *ra, rc, re, rf*) in each model on the power to reject g1 = 0, i.e. the causal parameter in the regression of trait 2 on trait 1 (see Fig. 1). This was performed for each model (DoC, MR-DoC, and MR-DoC2). We regressed the calculated NCPs (step 5, previous section) on the parameters’ true values. The resulting R^2^ statistics from these regressions represent the proportions of variance in the NCP explained by all the predictors, and the coefficients, the contributions of the individual predictors on the NCP variance. This allows us to gauge the effect of the parameters’ values on the model’s power. Note that all models were fit to data generated with MR-DoC2, which includes a second causal path (*g2*), the second instrument path (*b3*), as well as environmental background confounding (*re*) and the correlation of the instruments (*rf*). The coefficients of these regressions were calculated and plotted as stacked bar plots in Fig. 6. Since we tested only the hypothesis that g1 = 0, only *g1* and *b1* have shown an influence on the power.

All analyses were performed in R version 4.1.3 (R Core Team 2021) running on a Linux OS (Solus OS distribution version 4.3). Modelling was performed using OpenMx version 2.20.7 (Neale et al. 2016).

## Results

### Bias due to measurement error

Phenotypic measurement error has been shown to bias the causal parameter estimates of the DoC model (Heath et al. 1993). To assess the impact of phenotypic measurement error, we introduced unreliability to both the exposure (10%; i.e., reliability .9) and the outcome (30%; i.e., reliability .7). The estimates obtained from the simulations are shown in Figs. 2 and 4. In MR-DoC the causal path (*g1*) was underestimated given unreliable phenotypic measurement (Fig. 2). Measurement error did not affect the causal path estimates (*g1, g2*) in MR-DoC2 (Fig. 2). Therefore, MR-DoC2 showed better performance under the condition of measurement error of the phenotypes.

MR-DoC2 includes parameters that are not present in the other models, specifically *g2, re, rf*, and *b3*. When the data were generated using the MR-DoC2 model (Fig. 4), we found more severe bias in DoC and MR-DoC. The absence of the paths from MR-DoC2 in MR-DoC (re, rf, b3) and DoC (b1, b2), resulted in greater bias in the models’ estimates than error measurement.

### Bias due to environmental confounding

It has been previously noted that the causal estimates (*g1*), as obtained in the DoC model are biased if *re* is incorrectly fixed to zero (Rasmussen et al. 2019). The effect of the misspecification with respect to re in MR-DoC has not been previously explored. We set up simulations two and four to examine this, i.e., how the violation of the assumption that *re* = 0 affects the parameter estimates (Fig. 3, and 5). If *re* is truly positive (+ 0.3), the specification *re* = 0 resulted in an overestimation of the causal parameter g1 in both DoC and MR-DoC. Conversely, *g1* would be underestimated if *re*=−0.3, Fig. 3. However, when models were fitted to data generated with MR-DoC2, the bias in the parameter estimate was larger and more pervasive for DoC than for MR-DoC (Fig. 5). As expected, the causal (*g1, g2*) and pleiotropic (*b1, b3*) paths remain unbiased in the MR-DoC2 simulation, as this is the data generating model.

### Power

Finally, we compared the statistical power profiles of each model. For this step, we generated data using the MR-DoC2 model (Table 1, Design 3), and then fitted each model to these data. MR-DoC2 is a bidirectional model, but here we focus on the power to reject the hypothesis that *g1* = 0, at an alpha level of 0.05 with samples of 1000 MZ and 1000 DZ twin pairs. The NCPs from this power test were regressed on the parameter values, and the coefficients for each regression were plotted as a stacked bar plot in Fig. 6. The total R^2^, the proportion of NCP variance explained by the parameters equalled 0.60 in the DoC model, 0.60 in the MR-DoC model, and 0.95 in the MR-DoC2 model.. The longer the bars of a given parameter, the more NCP variance the parameter explains. We found that *g1* had the largest effect on DoC and MR-DoC power; and *g1,* and *b1* had large effects on the MR-DoC2 power. Also, *ra* and *rc* had small, but noticeable, effects on the power in the DoC and MR-DoC models. Note that, in MR-DoC, the path from the instrument to the exposure (*b1*) did not contribute to the power of the model. This means that the instrument R^2^ does not influence the power to reject the hypothesis that *g1* = 0 at the .05 significance level in MR-DoC. Furthermore, all power tests performed were under the hypothesis of *g1* = 0 in order to make the results more comparable between models. However, it is also possible to perform a 2 df power test in MR-DoC2 dropping both *g1* and *g2*. When this was done, the parameters *g2, b3* and *rf* also showed important influence in predicting the NCP variance (not shown).

## Discussion

We presented a series of simulations that address issues regarding: 1) measurement error, 2) misspecification of unshared environmental confounding, and 3) the statistical power of three models: DoC, MR-DoC, and MR-DoC2. We found that the models differ in how they are affected by issues 1 and 2, and in the role played by the instrumental variable(s) between MR-DoC and MR-DoC2. The estimates of the causal path (*g1*) were biased in the DoC and MR-DoC models when there was measurement error of the phenotypes, or when re was misspecified as equal to zero. We also found that the power profiles differed between the models. For MR-DoC2, *b1* and *g1* were the parameters that had the largest effects on power to reject the hypothesis that *g1* = 0, whereas in the DoC and MR-DoC the key parameters were *g1, ra* and *rc* (Fig. 6). We color-coded Fig. 1 to represent these results; the paths marked in blue have biassed estimates in the case of misspecification of re, the ones in red contribute relatively greatly to the NCP variance (power), and the ones in orange are important to power and are biassed.

When evaluating the power profile of MR-DoC, we found that the instrument strength does not explain any variance on the NCP to reject the false hypothesis of no causation. In other words, there is no requirement of a strong instrument in MR-DoC’s case. This is particularly important in classic MR, where the weaker the instrument, the greater the bias (Burgess et al. 2011).

MR-DoC explicitly includes horizontal pleiotropy in the parameter *b2*. It therefore is a model that addresses this problem directly, allowing causal inference unencumbered by the presence of horizontal pleiotropy. To identify the *b2* parameter it is necessary to assume that specific environmental confounding (re) is zero. It should be noted, however, that *b2* was slightly biased by unmodeled non-zero *re* (Fig. 2, green bar).

Parameter estimates from the MR-DoC2 model were consistently the least biased in all tests performed. The absence of re and pleiotropic paths like *rf* * *b1* or *rf* * *b3* in MR-DoC and in DoC biased the casual path estimate (*g1*). Another strength of MR-DoC2 is its feedback loop structure, thereby allowing inference regarding bidirectional causation. Feedback loops are frequent in nature, and most current MR methods cannot evaluate this type of relationship. This limitation is due to the acyclic nature of directed acyclic graphs, an assumption that does not affect maximum likelihood based inference. We recently reported one such example supporting a feedback loop between cigarettes per day and a maintenance factor, explained by the satiation resulting from smoking down-regulating the impulse for the next cigarette (Verhulst et al. 2021).

The tests presented also revealed an important aspect of model comparisons in SEM. Due to the non-independence of model parameters, changes to any of them will necessarily result in (sometimes severe) changes to other paths (Figs. 4 and 5). We also converted all three models to the variance component style in order to maintain coherence with our recent publications (Verhulst et al. 2019; Maes et al. 2022). The variance component style (as opposed to the more traditional RAM specification) does not inflate the Type II error (Verhulst et al. 2019). A practical advantage of this approach is that it is faster than the RAM specification.

This study should be interpreted in the light of the following limitations. The bias analysis and the results of the power analyses of these SEM models serve only as an aid to understanding how biases may arise and the power in specific scenarios considered. Changes to a single parameter in such models leads to changes in most other paths, making comparisons and interpretation not straightforward. For example, as noted above, setting *g2* = 0 in the power test revealed that *b3, rf*, and *g2* were influential to the NCP variance (not shown).

The models presented here overcome important limitations inherent in classical MR. MR-DoC does not require a strong instrument, and bidirectional causal inference is possible in the cross-sectional case. Both models (MR-DoC and MR-DoC2) explicitly include horizontal pleiotropy, which is an important limitation to causal inference in other MR methods. They open causal inference to interesting new possibilities whenever twin data are available, like true bidirectional causal inference or being able to test for causality unencumbered by horizontal pleiotropy.

## Figures and Tables

**Table 1:** Parameter values in the three factorial designs, with respective total number of cells for each design simulation. See Fig. 1 for the model specification. Also, $e_{1}$ was specified as $1-a_{1}-c_{1}$ and $e_{2}$ as $1-a_{2}-c_{2}$. Parameters x and y arenot listed, as they remained unchanged across the designs.

θ	Design 1 (DoC)	Design 2 (MR-DoC)	Design 3 (MR-DoC2)
b_1_		$\sqrt{0.025},\sqrt{0.05}$	$\sqrt{0.025},\sqrt{0.05}$
b_2_		$\sqrt{0.025},\sqrt{0.05}$	
b_3_			$\sqrt{0.025},\sqrt{0.05}$
g_1_	$\sqrt{0.02},\sqrt{0.04}$	$\sqrt{0.02},\sqrt{0.04}$	$\sqrt{0.02},\sqrt{0.04}$
g_2_			$\sqrt{0.02},\sqrt{0.04}$
ra	0,.2	0,.2	0,.2
rc	0,.2	0,.2	0,.2
re			.2
rf			.2
a_1_	.10	.10	.10
a_2_	.10	.10	.10
c_1_	.10	.10	.10
c_2_	.10	.10	.10
Total cells	2^3^=8	2^5^=32	2^6^=64

**Table 2:** Change in covariance when re = 0.3 (in contrast with re = 0) for the MR-DoC model.

	XI	Y1	PSxl	X2	Y2
**X1**	0.000	0.300	0.000	0.000	0.000
**Y1**	0.300	0.085	0.000	0.000	0.000
**PSx1**	0.000	0.000	0.000	0.000	0.000
**X2**	0.000	0.000	0.000	0.000	0.300
**Y2**	0.000	0.000	0.000	0.300	0.085

## Data Availability

Data sharing not applicable to this article as no datasets were generated or analyzed during the current study.
